# Is it congenital cystic eye with dermal appendages and cerebral anomalies?

**DOI:** 10.4103/0301-4738.67053

**Published:** 2010

**Authors:** V P Gupta, S N Bhattacharya, Pragati Gupta, Rigved Gupta

**Affiliations:** Department of Ophthalmology, University College of Medical Sciences and G.T.B. Hospital, Delhi - 110 095, India; 1 Department of Dermatology, University College of Medical Sciences and G.T.B. Hospital, Delhi - 110 095, India; 2 Postgraduate in Ophthalmology, LHMC, New Delhi, India; 3 University College of Medical Sciences and G.T.B. Hospital, Delhi - 110 095, India

Dear Editor,

We read the article by Arora *et al*.[1] with interest. We wish to make following observations:

1. The authors have described that the skin over the swelling was hypoplastic with prominent veins. However, there is no evidence of hypoplastic skin over the swelling in the clinical photograph. The dermatological findings reported by Delleman and Oorthuys in their four cases were periorbital skin appendages and focal dermal defects involving the supra- and retroauricular areas, the nose tip, philtrum, shoulder, cervical, inguinal region, thumb, trunk, and eyebrows.[2–4] They reported three varieties of focal dermal defects in the form of aplasia, hypoplasia, and punch-like defects.[2–4] The clinical appearance of focal dermal hypoplasia / aplasia could range from loss of skin appendages such as hair and glands, to focal depression of skin and visible subcutis (fat), and the healing of such a defect by secondary intention and scar formation. Except for multiple periorbital skin appendages none of these dermatological findings are visible in the photograph.
2. The constellation of findings in this case, that is, a large cystic swelling covered by the upper eyelid, with prominent veins, positive transillumination test, and a hypodense cystic area, with anophthalmia on computed tomography (CT), are pathognomonic of congenital cystic eye. Delleman and Oorthuys reported the presence of microphthalmos with an orbital cyst in their cases. None of their cases had congenital cystic eye. The minimal diagnostic criteria for this syndrome include a central nervous system (CNS) cyst or hydrocephalus, microphthalmia with orbital cyst, and focal dermal hypoplasia or aplasia.[5] As two of the components, that is, microphthalmia with orbital cyst and focal dermal hypoplasia or aplasia were missing from the report of Arora *et al*.[1] it did not appear to be a typical case of Delleman and Oorthuys syndrome. Congenital cystic eye could occur in isolation or with other malformations including, dermal appendages, agenesis of corpus callosum, and colpocephaly.[6] Other nonocular abnormalities that have been reported in association with congenital cystic eye include, facial clefting, saddle nose, nostril malformation, choanal atresia, malformation of the sphenoid bone, basal encephalocele, midbrain deformities, microphallus with encephalocele, hypoconvex fingernails on short stubby fingers, and bifid thumb.
3. The clinical photograph of the left eye at one year demonstrates a grossly swollen upper eyelid, which could be due to the recurrence of a cyst, despite ethanolamine oleate sclerotherapy. The authors should have shown the picture of both eyes for comparison. In view of repeated recurrence of cysts, excision of cystic eye with orbital implant and custom-made gradually expanding prosthesis is recommended.
